# Identification of potential functional variants and genes at 18q21.1 associated with the carcinogenesis of colorectal cancer

**DOI:** 10.1371/journal.pgen.1010050

**Published:** 2022-02-02

**Authors:** Xiaoqing Cheng, Fenglan Zhang, Jingwen Gong, Yige Li, Dan Zhou, Jing Wang, Eu Gene Vong, Ying Yuan, Maode Lai, Dandan Zhang

**Affiliations:** 1 Department of Pathology, and Department of Medical Oncology of the Second Affiliated Hospital, Zhejiang University School of Medicine, Hangzhou, Zhejiang, China; 2 Department of Pathology, Key Laboratory of Disease Proteomics of Zhejiang Province, School of Medicine, Zhejiang University, Hangzhou, China; 3 Department of Biochemistry and Genetics, Zhejiang University School of Medicine, Hangzhou, Zhejiang, China; 4 Department of Medical Oncology, the Second Affiliated Hospital, Zhejiang University School of Medicine, Hangzhou, Zhejiang Province, China; 5 Cancer Institute (Key Laboratory of Cancer Prevention and Intervention, Chinese National Ministry of Education), the Second Affiliated Hospital, Zhejiang University School of Medicine, Hangzhou, Zhejiang Province, China; Brigham and Women’s Hospital, UNITED STATES

## Abstract

Genome-wide association studies (GWAS) have identified more than 160 susceptibility loci for colorectal cancer (CRC). The effects of these variants, particularly their mechanisms, however, remain unclear. In this study, a comprehensive functional annotation of CRC-related GWAS signals was firstly conducted to identify the potential causal variants. We found that the SNP rs7229639 in intron 3 of *SMAD7* at 18q21.1 might serve as a putative functional variant in CRC. The SNP rs7229639 is located in a region with evidence of regulatory potential. Dual-luciferase reporter assays revealed that three other SNPs (rs77544449, rs60385309 and rs72917785), in strong linkage disequilibrium (LD) with rs7229639, exhibited allele-specific enhancer activity, of which one of the target genes may conceivably be *LIPG*, as suggested by eQTL association data and Hi-C data. We also verified that *LIPG* promoted malignancy of CRC cells in vitro, with supporting clinical data indicating that *LIPG* is upregulated and correlated with a poor prognosis in CRC. Finally, pitavastatin was observed to exhibit an anti-CRC activity and modest inhibition of *LIPG* mRNA levels. Collectively, our data suggest that these functional variants at 18q21.1 are involved in the pathogenesis of CRC by modulating enhancer activity, and possibly *LIPG* expression, thus indicating a promising therapeutic target for CRC. The results of functional annotation in our investigation could also serve as an inventory for CRC susceptibility SNPs and offer guides for post-GWAS downstream functional studies.

## Author summary

In the latest statistics, the incidence and mortality rate of colorectal cancer (CRC) remains high. Genome-wide association studies (GWAS) have become a powerful tool for identifying genetic susceptibility loci that confer significant risk on disease, and have identified more than 160 risk loci associated with CRC. However, it has proven quite difficult to identify the regulatory variants and target genes involved behind these GWAS signals. Here, we take advantage of multi-omics data and multiple biological experiments to reveal new biological pathways affecting susceptibility to CRC. We show that a specific genetic variant, rs7229639, and three other high linked functional variants (rs77544449, rs60385309 and rs72917785) at 18q21.1 might regulate the expression of *LIPG*, a gene that was shown to exhibit an oncogenic function by our in-vitro experiments and clinical data analysis. The link between genetic variants, gene expression and CRC phenotype established by us could provide references for follow-up basic and clinical studies.

## Introduction

Colorectal cancer (CRC) is one of the most common cancers worldwide, with significant morbidity and mortality [1]. The development of CRC is multifactorial, i.e., influenced by both environmental and genetic factors; while it is estimated that genetic factors contribute to over 30% of risk [2]. Thus far, genome-wide association study (GWAS) is the primary tool to reveal genetic susceptibility loci for complex diseases and traits that has unequivocally revolutionized the field of complex disease genetics over the past decade. Over 160 genetic loci associated with CRC have been identified by GWAS as of now, cumulatively explaining a substantial proportion of genetic risk involved in CRC [3–9]. Even so, the causal variant(s), the targeted gene(s), and the molecular mechanisms underlying these associations have yet to be fully understood.

One of the major challenges to understand the functional basis of these associations might be that many of GWAS-identified single-nucleotide polymorphisms (SNPs) are located in noncoding regions and not necessarily exert their effects via regulating the nearest genes [10]. Emerging evidence suggests that causal SNPs tend to be in linkage disequilibrium (LD) with the corresponding tag SNP, and are encompassed within potential regulatory elements (PREs) that control distal gene expression through long-range genome interactions [11–13]. To effectively translate GWAS findings into references for downstream functional work, a preliminary understanding of these identified variants through functional annotation might be a better strategy to locate the possible gene targets, and to explore the potential mechanisms by which the variants influence the risk of a disease.

The recent emergence of large, publicly available databases containing extensive genomic and epigenomic data provides a wealth of annotation resources for characterizing these risk SNPs. For instance, the Genotype-Tissue Expression (GTEx) Project assembled a comprehensive data of expression quantitative trait loci (eQTLs) for dozens of cell and tissue types [14–16], while the Encyclopedia of DNA Elements (ENCODE) Project [17], the Roadmap Epigenomics Project [18] and others feature a plethora of annotated putative regulatory elements across hundreds of human cell types and tissues, as well as defining variants that are associated with histone modification (hQTLs). In addition, previous studies have also associated genetic variants with DNase I hypersensitivity (dsQTLs) [19] and transcription factor (TF)-binding (bQTLs) characteristics [20] which could alter the activity of cell type-specific regulatory elements, with ensuing changes in target gene expression.

**Fig 1:**
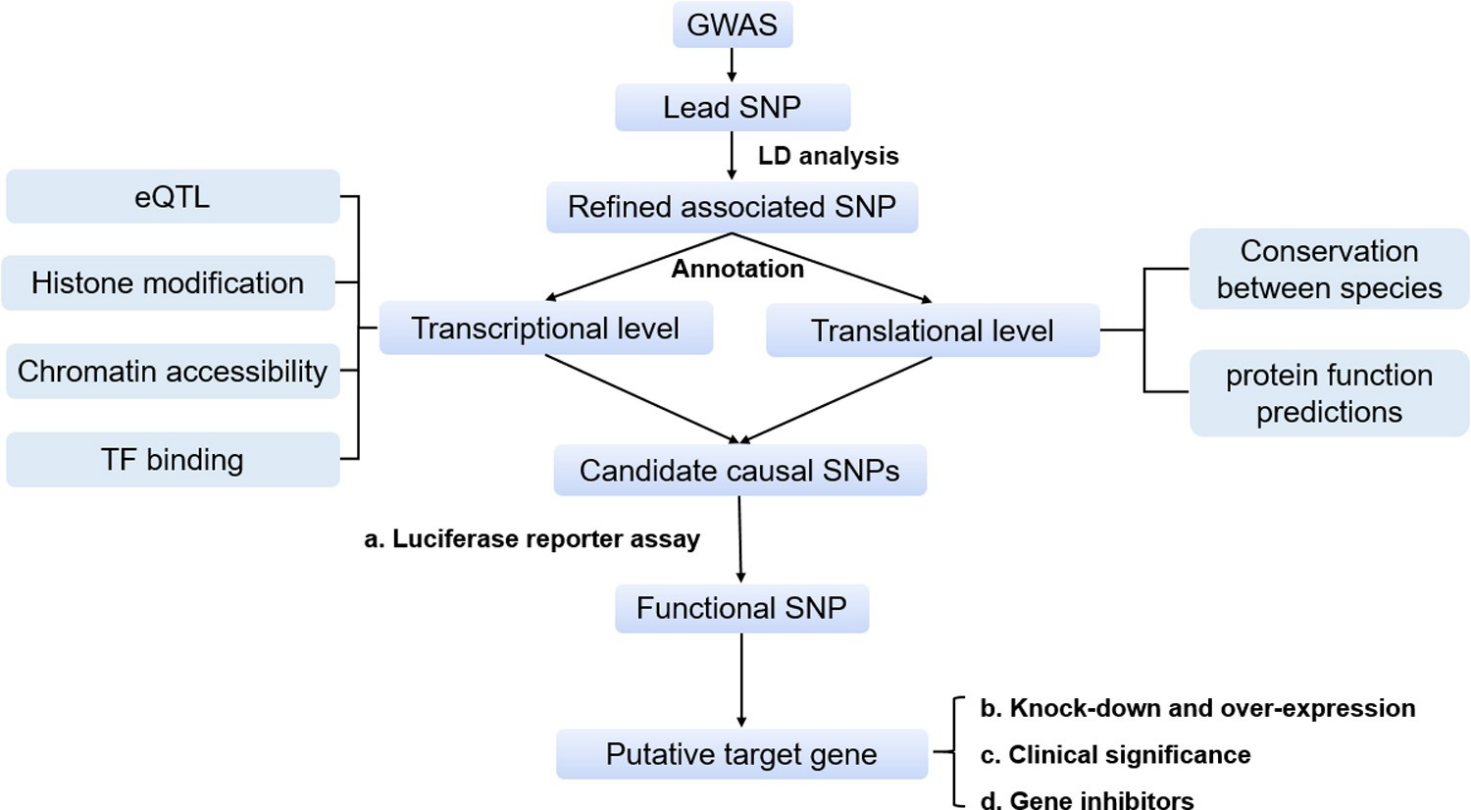
Flowchart of the comprehensive analysis strategy. CRC-related lead SNPs were obtained from the NHGRI-EBI GWAS catalog. LD calculation was performed to acquire SNPs in high LD with the lead SNPs (called refined associated SNPs) using the 1000 Genomes phase 3 dataset as the reference panel. Comprehensive functional annotation towards these SNPs was conducted at both the transcriptional and translational level to prioritize candidate causal SNPs. Then, various experimental methods were used to identify the functional SNPs and their putative target genes, such as reporter assays.

To identify causal variants and elucidate the functional mechanisms underlying CRC GWAS signals, we conducted a systematic functional annotation for CRC-related GWAS hits using multi-omics data, followed by a series of functional validation experiments; represented in the flowchart shown in **Fig 1**. We found that three SNPs at 18q21.1 (rs77544449, rs60385309 and rs72917785), all in strong LD with rs7229639, affect enhancer activity, and potentially the expression of *LIPG*, which encodes endothelial lipase (EL), hence contributing to CRC risk. By establishing the association between genetic variants, gene expression and disease, our findings provided valuable clues for subsequent basic and clinical studies.

## Results

### Functional annotation for CRC-related GWAS hits based on multi-omics data

After querying and filtering, 356 CRC-associated SNPs were included from the NHGRI-EBI GWAS Catalog (May 2019, S1 Fig), which were assigned as the lead SNPs. LD calculation was conducted using PLINK according to the 1000 Genomes phase 3 dataset, and we acquired 9,255 SNPs in high LD with the lead SNPs (S1 Fig, *r*^2^ > 0.5).

Subsequently, we performed comprehensive functional annotation for all susceptibility loci. According to eQTL results derived from the GTEx Project [15], 36 out of 356 lead SNPs were observed to affect gene expression levels in sigmoid or transverse colon tissues (S1 Table). For these eQTL SNPs, we further explored the possibility of influencing other molecular phenotypes, including chromatin accessibility, enrichment of histone marks, and TF binding. Of the 36 eQTL SNPs, we found that 16 were strongly linked with SNPs associated with DNase I hypersensitivity sites (*P* < 0.05, S2 Table). Histone modification enrichment revealed that 34 out of the 36 eQTL SNPs were in high LD with one or more SNPs enriched in histone marks (H3K27ac, H3K4me1 and H3K4me3) among various tissues (S3 Table). Additionally, 21 out of the 36 eQTL SNPs were in high LD with SNPs which have the potential to disrupt TF binding sites (*P* < 0.05, S4 Table). We finally explored the potential effects of the missense SNPs using SIFT and PolyPhen-2. Of 9,255 refined associated SNPs, we identified 44 SNPs that encoded missense substitutions, and 7 SNPs were predicted to be damaging through at least one algorithm (S5 Table). These results may serve as an inventory for CRC susceptibility SNPs and offer guides for downstream functional experiments.

### Candidate regulatory variants located in the *SMAD7* gene at 18q21.1

**Fig 2:**
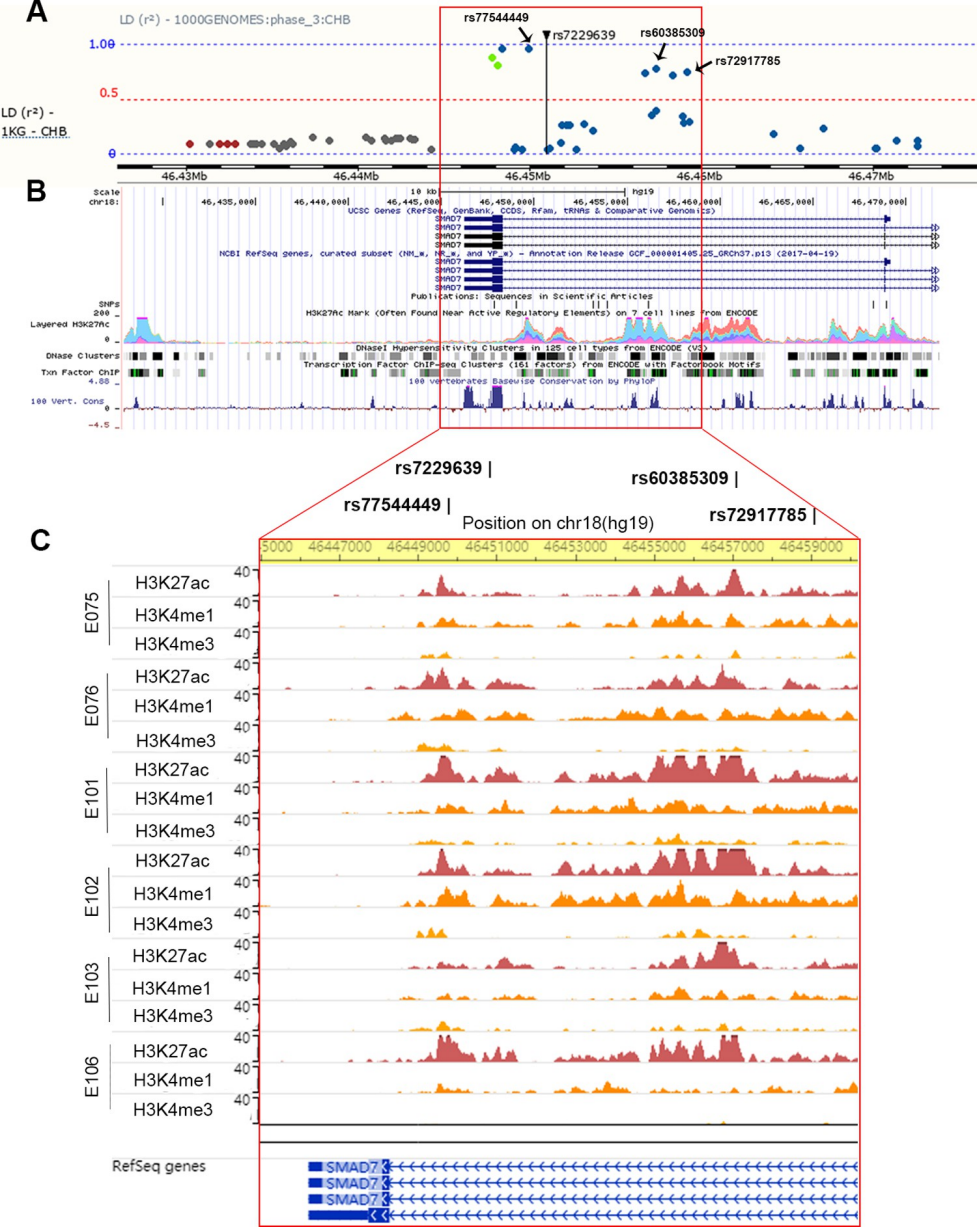
Overview of the genomic and epigenetic profiling in the LD region of rs7229639. (**A**) LD Manhattan plot of rs7229639 in CHB population (1000 Genomes phase 3) showed eight additional variants in high LD with rs7229639 (*r*^2^ > 0.5), including rs77544449, rs60385309, and rs72917785. The data were obtained from the Ensembl Genome Browser (v96). (**B**) Epigenetic annotation for LD region of rs7229639, including active histone modification H3K27ac, DNase I hypersensitivity and TF binding sites. These data were obtained from the ENCODE Project database and visualized by UCSC genome browser. (**C**) Tissue-specific histone modification signals (H3K27ac, H3K4me1 and H3K4me3) of the LD region of rs7229639 in six tissues, including Colonic Mucosa (E075), Colon Smooth Muscle (E076), Rectal Mucosa Donor 29 (E101), Rectal Mucosa Donor 31 (E102), Rectal Smooth Muscle (E103), and Sigmoid Colon (E106). The data were taken from the Roadmap Epigenomics Project and visualized by the WashU Epigenome Browser.

By conducting a comprehensive functional annotation for all the CRC susceptibility loci, we found that rs7229639, located in intron 3 of the *SMAD7* gene at 18q21.1, might serve as a candidate functional SNP in CRC. Its association with CRC risk was reported by five GWASs of East Asians [4,6–9]. The eQTL results from the GTEx Project show that among surrounding genes of the risk SNP rs7229639 (approximately 1 Mb upstream and downstream), only the *LIPG* gene, located > 636 Kb downstream, is significantly associated with rs7229639-A allele (risk allele) in sigmoid colon tissues (S2A Fig and S1 Table). Besides, we validated this correlation in the HapMap Asian populations (CHB and JPT), and the result was in line with the data from the GTEx project (S2B Fig). We investigated the long-range chromatin interactions between the *SMAD7* locus and *LIPG* using various published Hi-C datasets visualized by 3D Genome Browser, and found that the *SMAD7* locus had potential long-range chromatin interactions with *LIPG* in small bowel tissues (S3 Fig). As displayed in **Fig 2A**, there were eight additional SNPs in high LD with rs7229639 (*r*^2^ > 0.5, CHB). Moreover, the evolutionarily conserved LD region containing rs7229639 overlaps TF binding sites in an open chromatin site, which is marked by DNase I hypersensitivity and high enrichment of histone modification (Fig 2B). Tissue-specific histone modification datasets from the Roadmap Epigenomics Project confirmed that the region overlaps many enhancer marks, including H3K27ac and H3K4me1, in colon and rectal tissues, whereas the signal of H3K27ac was much weaker at the region in other tissues except for liver (Figs 2C and S4). Among the eight SNPs, rs77544449, rs60385309, and rs72917785 (*r*^2^ = 0.96, 0.78, and 0.76 with rs7229639, respectively; CHB) are the most promising functional candidates because of their location with evidence of regulatory potential (i.e., TF binding, DNase I hypersensitivity and active histone modification).

### Evaluation of allele-specific enhancer activity of candidate functional SNPs

**Fig 3:**
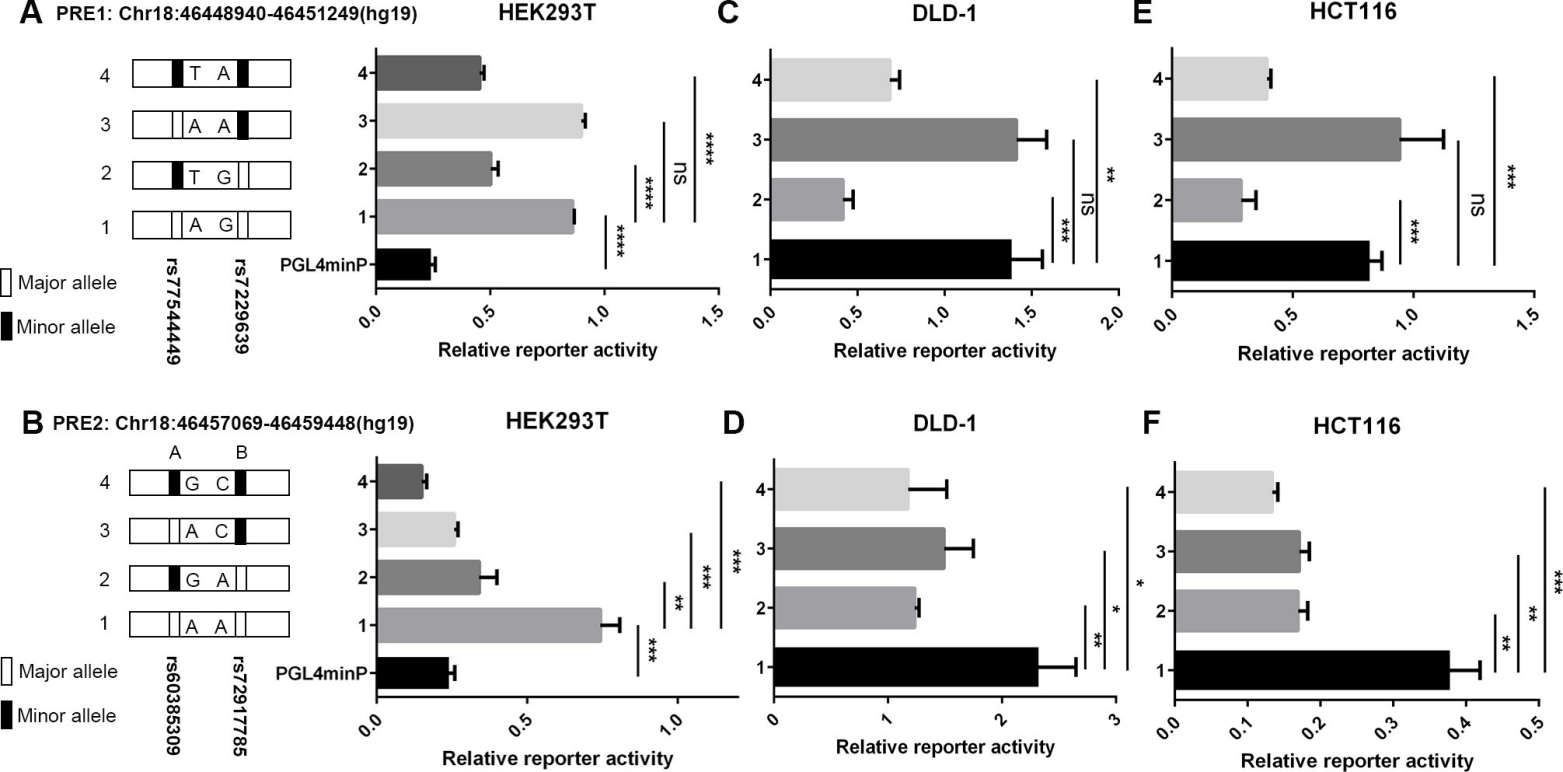
Luciferase reporter assays of two PREs containing candidate functional SNPs. (**A**) Luciferase reporter assays of PRE1 containing rs77544449 and rs7229639 with either the major or minor allele, or empty vector (PGL4minP) were performed in HEK293T cells. (**B**) Luciferase reporter assays of PRE2 containing rs60385309 and rs72917785 with either the major or minor allele, or empty vector (PGL4minP) were performed in HEK293T cells. (**C-F**) Luciferase reporter assays of two PREs containing candidate functional SNPs with either the major or minor allele was measured in DLD-1 cells or HCT116 cells. Two-tailed Student’s t tests were used to calculate *P* values. Error bars, SD. n = 3. * *P*<0.05, ** *P* < 0.01, *** *P* < 0.001, **** *P* < 0.0001. ns, not significant.

As described above, we observed that the rs7229639-containing LD block showed strong enrichment of H3K27ac and H3K4me1, of which both are hallmarks of enhancers. This suggested to us that the candidate causal variants might contribute to CRC risk by perturbing enhancer activity and consequently interfering with the target gene expression. Using epigenetic data from the ENCODE Project and the Roadmap Epigenomics Project (Fig 2B and 2C), we first identified two PREs that each harbored two candidate functional SNPs (PRE1: chr18:46448940–46451249, containing rs77544449 and rs7229639; PRE2: chr18:46457069–46459448, containing rs60385309 and rs72917785; hg19). We then cloned the two PREs with the major or minor allele of the corresponding SNP into pGL4.23 plasmids bearing the minimal promoter (minP) and thereafter measured luciferase activity in HEK293T cell lines. The two PREs exhibited strong transcriptional activity compared to the empty control vector (*P* < 0.001, **Fig 3A and 3B**), consistent with the predicted enhancer activity. Importantly, there was no significant difference in enhancer activity between the major or minor allele of rs7229639 (Fig 3A), whereas a significant decrease in luciferase expression was detected between the major and minor allele of the other three SNPs in high LD with rs7229639 (Fig 3A and 3B; *P* < 0.0001 for rs77544449; *P* < 0.01 for rs60385309; *P* < 0.001 for rs72917785, respectively), with the major alleles responsible for the enhancer activity, an effect opposite to the eQTL results of the *LIPG* gene (S2 Fig). The impact on the luciferase expression was distinctively strengthened when two SNPs within the same PRE were both mutated (Fig 3A and 3B). Consistent results were also obtained in two CRC cell lines (Fig 3C–3F). Together, these data indicated that the two regions encompassing variants may function as enhancer elements with allele-specific activity.

The allele-specific activity of two PREs might be due to the differences in TF binding to alleles of these functional SNPs. We conducted motif analysis using multiple tools and identified three motifs, RREB1, ELF3 and ZNF263, as candidate factors preferably binding to rs77544449-A, rs60385309-G and rs72917785-C, respectively (S5 Fig).

### *LIPG* promotes proliferation, migration and invasion of colorectal cancer cells

**Fig 4:**
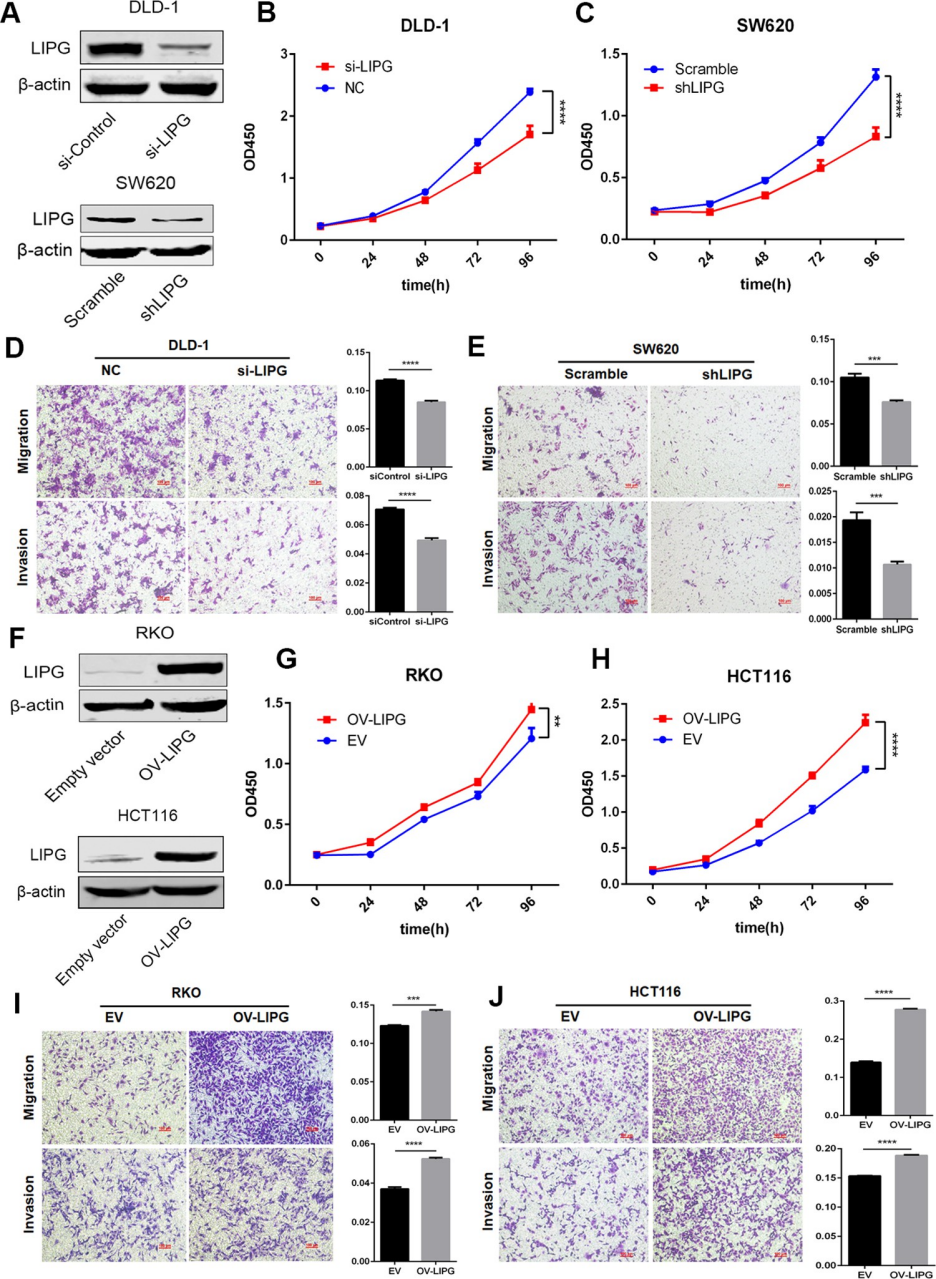
*LIPG* promotes CRC cell proliferation, migration and invasion in vitro. (**A**) Validation of *LIPG* knockdown efficiency by immunoblotting analysis in DLD-1 and SW620 cells. (**B, C**) The effect of *LIPG* knockdown on cell proliferation was measured by CCK-8 assay. (**D, E**) The effect of *LIPG* knockdown on cell migration and invasion capacity was measured by Transwell assay. (**F**) Validation of *LIPG* overexpression efficiency by immunoblotting analysis in RKO and HCT116 cells. (**G, H**) The effect of *LIPG* overexpression on cell proliferation was measured by CCK-8 assay. (**I, J**) The effect of *LIPG* overexpression on cell migration and invasion capacity was measured by Transwell assay. Error bars, SD. n = 3. Two-tailed Student’s t tests were used to assess statistical significance. ** *P* < 0.01, *** *P* < 0.001, **** *P* < 0.0001.

As mentioned above, *LIPG* might be the target gene of the two PREs. We examined the effect of *LIPG* knockdown or overexpression on cellular phenotypes. When *LIPG* was knocked down in DLD-1 and SW620 cell lines (both with high basal levels of *LIPG* expression) (**Figs 4A** and S6), the proliferation capacity was significantly inhibited in both cell lines (Fig 4B and 4C). Transwell migration/invasion assays revealed that the loss of *LIPG* expression decreases the potential of motility and invasive properties in both cell lines (Fig 4D and 4E). The consistent changes of cell phenotypes were obtained with another siRNA sequence targeting *LIPG* (S7 Fig). In contrast, overexpression of *LIPG* in RKO and HCT116 cells (both with low basal levels of *LIPG* expression) resulted in the promotion of cell proliferation, migration and invasion (Figs 4F–4J and S6).

### Clinical impact of *LIPG* expression on CRC progression

**Fig 5:**
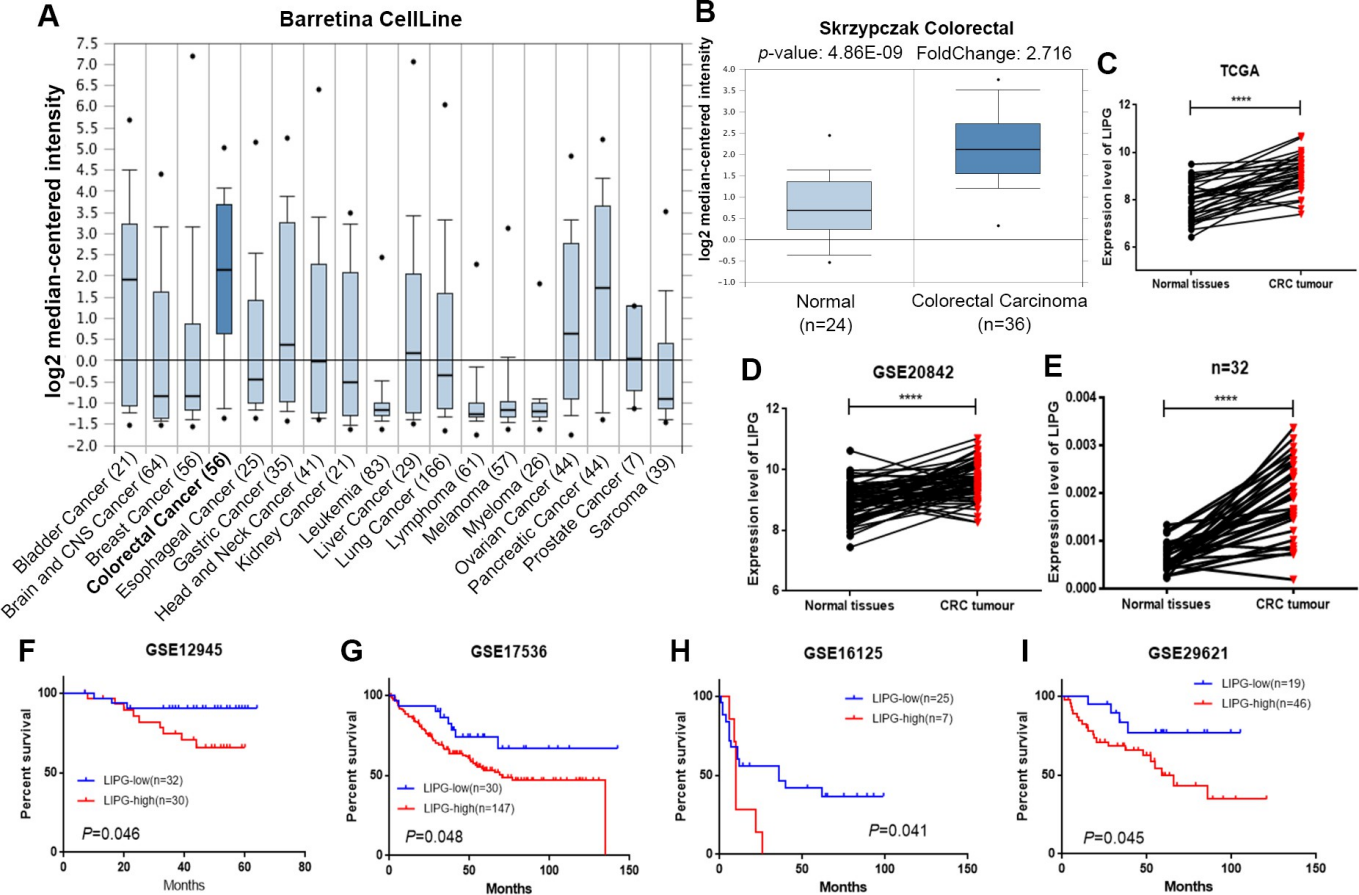
*LIPG* mRNA levels are upregulated in CRC and associated with poor prognosis. (**A**) *LIPG* was distinctly expressed higher in CRC, compared with other carcinoma types from the Oncomine Database (Barretina CellLine). (**B–E**) Relative expression of *LIPG* in CRC and adjacent or normal tissue samples from the Oncomine Database (B), TCGA (C), GEO GSE20842 dataset (D), and our own CRC samples (E). (**F–I**) Kaplan-Meier plots showed that high expression of *LIPG* was associated with poor prognosis in multiple GEO datasets, including GSE12945 (F), GSE17536 (G), GSE16125 (H), and GSE29621 (I). Paired samples were assessed by paired t-test, otherwise unpaired t-test was used. **** *P* < 0.0001.

By querying the Oncomine Database, *LIPG* was shown to be highly expressed in CRC, as compared to other types of cancer (**Fig 5A**). To evaluate the clinical significance of *LIPG* expression in CRC, we first investigated the differences in expression of *LIPG* between CRC and adjacent or normal tissue samples from the Oncomine Database, TCGA, the GEO GSE20842 dataset and our own CRC samples. The results showed that the mRNA levels of *LIPG* are significantly higher in CRC cancer tissues (Fig 5B–5E). We further evaluated the prognostic value of *LIPG* in four GEO datasets (GSE12945, GSE17536, GSE16125 and GSE29621) and found that *LIPG* upregulation was associated with poor prognosis in CRC patients (Fig 5F–5I). Finally, we explored the correlations between *LIPG* expression and clinicopathological factors in TCGA Colon Adenocarcinoma (COAD) and Rectum adenocarcinoma (READ) datasets. *LIPG* expression was correlated with histological classification (*P* = 0.006, FDR = 0.024), lymph node metastasis (*P* = 0.018, FDR = 0.048) and TNM stage (*P* = 0.003, FDR = 0.024). All association data with clinicopathological features of CRC patients are summarized in S6 Table. Overall, these clinical data strongly associate *LIPG* expression with CRC development.

### Effect of pitavastatin on colorectal cancer cells and *LIPG* expression

**Fig 6:**
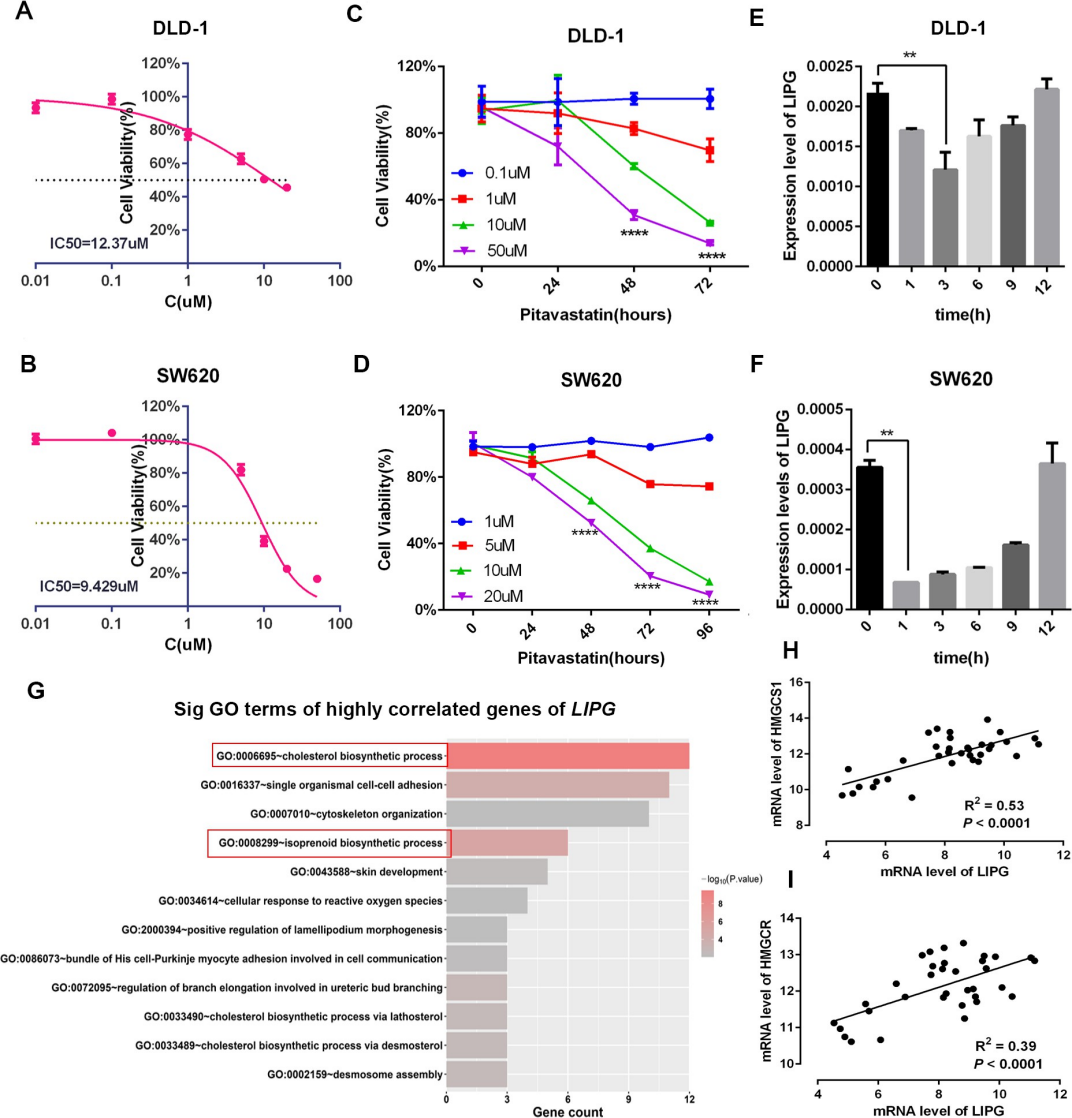
Effect of pitavastatin on CRC cell viability and *LIPG* mRNA expression. (**A, B**) IC_50_ fitting curve for pitavastatin applied to DLD-1 and SW620 cells at 48 h. (**C, D**) Effect of pitavastatin on cell viability of DLD-1 and SW620. (**E, F**) Effect of pitavastatin treatment on *LIPG* mRNA levels in DLD-1 and SW620 cells. Error bars, SD; two-tailed Student’s t tests. (**G**) Top enriched biological process terms related to *LIPG* identified via gene ontology enrichment analysis of the highly correlated genes of *LIPG* based on DAVID online tools (*P* < 0.01). (**H, I**) The correlations between the mRNA levels of *LIPG* and those of *HMGCS1* (H) or *HMGCR* (I) in 34 CRC cell lines (GSE97023). ** *P* < 0.01, **** *P* < 0.0001.

In view of the promotive effect of *LIPG* on CRC progression, inhibition of *LIPG* expression might be a novel approach for CRC therapy. Pitavastatin, a blood cholesterol-lowering drug of the statin class, had previously been reported to decrease the expression of EL, which is encoded by *LIPG* [21]. Thus, we explored whether the existing drug would exhibit anti-CRC effects via inhibiting *LIPG* expression. We first evaluated the cytotoxicity effect of the drug on CRC cells and found that pitavastatin could kill DLD-1 and SW620 cells in a dose-dependent manner, with the IC_50_ concentration in both cell lines being relatively low (**Fig 6A and 6B**, 12.37 μM in DLD-1; 9.43 μM in SW620). Moreover, the inhibitory rate of pitavastatin appears to increase with extended duration of treatment (Fig 6C and 6D). Next, we assessed the effect of pitavastatin on *LIPG* mRNA levels in DLD-1 and SW620 cell lines. *LIPG* mRNA levels were observed to be inhibited transiently after treatment with pitavastatin (Fig 6E and 6F).

To further link the effect of pitavastatin to *LIPG*, we explored whether there was a connection between the biological function of *LIPG* and pitavastatin. We identified the 500 most highly co-expressed genes with *LIPG* by gene expression correlation analysis utilizing the GSE97023 dataset (n = 34), upon which genome-wide expression analyses on 34 colorectal cancer cell lines were performed. Gene ontology (GO) analysis showed that these genes were enriched in cholesterol biosynthetic process and isoprenoid biosynthetic process (also referred to as the HMG-CoA reductase pathway) (Fig 6G). *LIPG* mRNA levels correlated with those of HMG-CoA synthase 1 (*HMGCS1*) and HMG-CoA reductase (*HMGCR*) gene encoding rate-limiting enzymes of cholesterol synthesis pathway (Fig 6H and 6I).

## Discussion

Despite GWASs have identified many genetic variants associated with CRC, little is known about the causal variants and the responsible pathogenic genes, and further functional studies are still very limited. Here, through a comprehensive functional annotation for all the CRC susceptibility loci obtained from GWASs, followed by functional experiments, we observed that three plausible functional variants (rs77544449, rs60385309 and rs72917785), which were in high LD with the risk SNP rs7229639, had allele-specific effects on enhancer activity, thus possibly modulating the expression of *LIPG*. Furthermore, in vitro experiments and clinical data analysis demonstrated that *LIPG* plays a non-negligible role in CRC cellular transformation and tumor progression. Our studies strongly point to the fact that upregulation of *LIPG* is one of the key factors accounting for the association between rs7229639 and CRC susceptibility.

Several studies have investigated the functional variants within enhancer elements in *SMAD7* intron 4 [22,23]. Pittman et al. implicated a novel SNP (novel 1 or rs58920878) as a functional change leading to CRC predisposition by altering *SMAD7* expression [23]. Fortini et al. reported that the associated CRC risk at 18q21.1 was due to four SNPs including novel 1 (rs6507874, rs6507875, rs8085824, and rs58920878) in an enhancer affecting the expression of *SMAD7* [22]. These SNPs are all in LD with rs4939827, a risk variant identified previously in a European-ancestry GWAS in relation to CRC risk [24]. In this study, after comprehensively characterizing all CRC susceptibility loci, we prioritized a noncoding SNP (rs7229639), which is located in intron 3 of the *SMAD7* gene. The SNP rs7229639 and rs4939827 are not correlated in East Asians (*r*^2^ = 0.008) nor in Europeans (*r*^2^ = 0.146). The association between rs7229639 and CRC remains statistically significant after adjusting for rs4939827 [9]. Furthermore, eQTL data from the GTEx Project showed an association between the risk allele rs7229639-A and increased expression of *LIPG*, but not *SMAD7*, in normal sigmoid colon tissues.

Recent studies have elucidated the intriguing fact that functional SNPs are located in PREs to regulate expression of distal gene by long-range interaction [10,12,25]. Properties such as overlapping with accessible chromatin, possession of TF binding sites, and specific histone marks for active regulatory activity might be evidences indicating the existence of a PRE and thus, a functional genomic region [26]. The pattern of histone modifications observed at the rs7229639-containing region was associated with enhancer activity, thus we proposed that this region might serve as an enhancer with allele-specific activity. In agreement with this hypothesis, our luciferase reporter assays showed that this region contained two PREs that promoted luciferase gene expression. However, except for rs7229639, only the other three SNPs (rs77544449, rs60385309 and rs72917785) demonstrated altered allelic activity. Our results revealed that the minor allele of these candidate functional SNPs showed lower enhancer activity than the major allele in the reporter assay. This result contrasts with the eQTL results linking the risk allele to increased *LIPG* expression. One possibility might be that there exist other regulatory elements in this region that could regulate *LIPG*. In addition, the discrepancy might be partially attributable to the limitations of reporter assays per se. Reporter assays are performed using plasmid DNA in vitro, not in the genomic context in which the SNP actually exists [27].

The evidences provided by our present study clearly pointed to *LIPG* as a plausible causative gene in CRC, consistent with the eQTL analyses that the risk allele rs7229639-A was associated with elevated *LIPG* expression. As a member of the triglyceride lipase family and a cell surface-associated lipase with predominantly phospholipase A1 activity [28], endothelial lipase plays a critical role in lipoprotein metabolism, in particular, high-density lipoprotein (HDL) metabolism [29]. Aberrant expression of *LIPG* was found in several cancer types, including breast cancer [30], gastric cancer [31] and testicular germ cell tumors [32]. *LIPG* has been reported to support growth and survival of cancer cells by mediating lipid metabolism and/or adaptation to oxidative stress in breast cancer [33,34]. Similarly, it is possible that the functional SNPs could contribute to CRC risk by increasing *LIPG* expression to supply lipid precursors during tumorigenesis.

Since *LIPG* could promote CRC progression, pharmacologic inhibition of *LIPG* expression is assumed to become a valuable therapeutic strategy for CRC. A previous study has shown that pitavastatin could decrease the expression of EL [21] and statins have been shown to reduce the risk of CRC [35]. Our results showed that pitavastatin induced significant inhibition of CRC cell proliferation but a modest decrease of *LIPG* mRNA levels at a given concentration. Gene expression correlation analysis further indicated that *LIPG* might be involved in HMG-CoA reductase pathway, which is best known as the target of statins [36]. Therefore, pitavastatin might have tumor suppressive effects by modulation of *LIPG* expression in CRC. A potential role for pitavastatin in the inhibition of stem cell proliferation in colon carcinoma has been reported in a direction consistent with our findings [37]. Not only that, the drug was also reported to have an inhibitory effect on liver cancer cells [38]. It should be noted, however, that the possible links between pitavastatin and other genes (except *LIPG*) have yet to be ruled out. Therefore, more work is required to prove that *LIPG* mediates the inhibitory effect exerted by pitavastatin on CRC cell viability, and a feasible method would be patient-derived tumor xenograft (PDX) models.

In addition, as with the majority of studies, the approach of our current study is subject to limitations and challenge. Firstly, due to the ever-constant updates, emergence and availability of annotation resources, our functional annotation for susceptibility loci may have missed out some novel information published as of late. Secondly, there might exist other functional SNPs regulating *LIPG* at this locus, which will need additional investigation to establish a direct or significant link on CRC cell growth through *LIPG*.

In summary, through comprehensive functional annotation, in vitro experiments and clinical data analysis, we have studied the roles of CRC-related genetic variants at 18q21.1 and their possible target gene, *LIPG*, in the development of CRC. Last but not least, pitavastatin is compellingly observed to antagonize the CRC promoting activity of *LIPG*. Our findings could provide clues to fine-mapping and functional validation for causative variants contributing to disease risk as well as hints to the prevention and treatment of CRC.

## Materials and methods

### Ethics statement

Ethical approval was obtained from the ethics committee of Sir Run Run Shaw Hospital, an affiliate of Zhejiang University, Medical College (number: 20190628–24). Thirty-two matched CRC and normal tissue samples were recruited from the hospital from 2004 to 2011. Written informed consents were obtained from all of the patients included.

### Comprehensive functional annotation for CRC susceptibility SNPs

As shown in S1 Fig, CRC-related SNPs with *P* ≤ 5 × 10^−5^ were retrieved from the NHGRI-EBI GWAS Catalog (May 2019; https://www.ebi.ac.uk/gwas/home) [39]. After excluding the SNPs with irrelevant or imprecise phenotypes (e.g., cancer, severe skin toxicity response to cetuximab in colorectal cancer, etc.), a total of 356 lead SNPs were selected for subsequent analyses. We obtained the SNPs in LD (*r*^*2*^ > 0.5) with the lead SNPs by utilizing the 1000 Genomes phase 3 dataset as the reference panel. Then, tissue-specific eQTL signals derived from the GTEx project (v7; https://www.gtexportal.org/home/) were employed to investigate whether the lead SNPs could affect gene expression in sigmoid and transverse colon tissues [14,15]. The significance level threshold for eQTL effects was described in detail in the paper of the GTEx project [16]. Specifically, variants with a nominal *P* value below the gene-level threshold were considered significant by the GTEx Consortium. We annotated epigenetic regulatory features for these eQTLs and their adjacent SNPs in LD using dsQTL data with a nominal *P* value < 0.05 from Grubert et al. (http://mitra.stanford.edu/kundaje/portal/chromovar3d/QTLs/localQTL/) [19] and histone modification data (H3K27ac, H3K4me1, and H3K4me3) in six tissues (E075-Colonic Mucosa, E076-Colon Smooth Muscle, E101-Rectal Mucosa Donor 29, E102-Rectal Mucosa Donor 31, E103-Rectal Smooth Muscle, and E106-Sigmoid Colon) from the Roadmap Epigenomics Project (v9; http://www.roadmapepigenomics.org/) [18]. Grubert and colleagues identified QTLs using DNase-I hypersensitivity (DHS) data from Degner et al. [40], who measured chromatin accessibility in 70 Yoruba lymphoblastoid cell lines (LCLs). For these dsQTLs, the direction of the regression coefficient (beta value) represents the effect of each extra major allele (i.e., a positive regression coefficient means that the major allele increases chromatin accessibility). Following this, we determined whether these SNPs have effects on TF binding (called as bQTLs) using the data generated from Tehranchi et al., who performed ChIP-seq for five TFs critical for immune cell development and function (NF-κB, PU.1, Stat1, JunD, and Pou2f1) in 60 Yoruba lymphoblastoid cell lines [20]. For SNPs in coding regions, we predicted the potential effect of missense SNPs on protein functions using SIFT (https://sift.bii.a-star.edu.sg/) [41] and PolyPhen-2 (http://genetics.bwh.harvard.edu/pph2/) [42].

### Identification of candidate regulatory variants and PREs

We retrieved the genotype data of rs7229639 (https://ftp.ncbi.nlm.nih.gov/hapmap/genotypes/) and *LIPG* expression data (GSE6536) of Asian populations (CHB and JPT) from the HapMap Project to validate the association of rs7229639 with *LIPG* [43]. Hi-C data on small bowel tissues were visualized by 3D Genome Browser (http://3dgenome.fsm.northwestern.edu/) [44,45]. The SNPs in LD with rs7229639 (*r*^*2*^ > 0.5, CHB) were downloaded from the Ensembl Genome Browser (v96; https://asia.ensembl.org/index.html) [46]. Then, regulatory features from Ensembl, epigenetic contexts from the ENCODE Project (ENCODE 4; https://www.encodeproject.org/) [17] and the Roadmap Epigenomics Project were combined to identify candidate regulatory variants in the LD region of rs7229639. The epigenetic data from ENCODE were displayed using the UCSC Genome Browser (http://genome.ucsc.edu/) [47]. Meanwhile, the tissue-specific histone modification profiles from the Roadmap Epigenomics Projects were displayed using the WashU EpiGenome Browser (https://epgg-test.wustl.edu/) [48]. Lastly, variants with the highest potential were selected for further experimental validation. Two PREs of length 2.3kb were identified based on the aforementioned epigenetic data.

### Cell lines

The human CRC cell lines (DLD-1, SW620, RKO, HCT116, and HCT8) and the human embryonic kidney 293T cells (HEK293T) were obtained from the American Type Culture Collection (ATCC). DLD-1, SW620, HCT116 and HCT8 were cultured in RPMI 1640 medium (HyClone) supplemented with 10% fetal bovine serum (FBS) (HyClone) and antibiotics (penicillin and streptomycin, Sigma). RKO and HEK293T were cultured in DMEM medium (HyClone) supplemented with 10% FBS and antibiotics. All cell lines were maintained at 37°C in 5% CO_2_ and free of mycoplasma. The cell lines used in this study were authenticated by short tandem repeat profiling.

### Dual-Luciferase reporter assay

The two 2.3 kb PRE fragments were amplified from genomic DNA with the primers listed in S7 Table and inserted into the upstream of the minimal promoter (minP) at the XhoI/HindIII restriction sites on the pGL4.23 [luc2/minP] reporter vector. Site-directed mutagenesis was performed to obtain either the major or minor allele of the four SNPs using the Mut Express II Fast Mutagenesis Kit V2 (Vazyme). Each recombinant or empty pGL4.23 vector was co-transfected into HEK293T cells along with pRL-TK vector containing Renilla luciferase with LipoD293 (SignaGen). Plasmids were transfected into CRC cell lines with Lipofectamine 2000 (Invitrogen). After 48 h, cell lysates were assayed for luciferase activity with the Dual-Luciferase Reporter Assay System (Promega) according to protocols recommended by the manufacturer. The luciferase activity was calculated by normalizing firefly luciferase expression to that of Renilla luciferase. The experiment was then replicated for three times. Measures were obtained from three replicates each time.

### Motif analysis

The effect of candidate functional variants (rs77544449, rs60385309 and rs72917785) on transcription factor binding motifs was analyzed using HaploReg v4.1 (https://pubs.broadinstitute.org/mammals/haploreg/haploreg.php) [49] and MEME Suite toolkit (https://meme-suite.org/meme/meme_5.3.2/doc/overview.html) [50] with TF motifs available from two public motif databases: JASPAR [51] and SwissRegulon [52]. Motifs with at least two hits by different databases were reported.

### qRT-PCR and immunoblotting

Total RNA was extracted from cell lines or tissues with TRIzol reagent (Invitrogen) and reversely transcribed with the PrimeScript RT Reagent Kit (TaKaRa). Then, qRT-PCR was performed with SYBR Premix Ex Taq (TaKaRa). Each gene expression was normalized against that of β-actin. All primers used are listed in S7 Table. For protein blot analysis, total protein was collected using RIPA lysis buffer supplemented with protease inhibitor cocktail (Roche). Protein were incubated with antibodies against *LIPG* (1:500, Abcam, Cat#ab24447), Flag (1:2,000, Sigma, Cat#F1804), or β-actin (1:5,000, CST, Cat#3700) overnight at 4°C.

### siRNA transfection

siRNA oligonucleotides targeting *LIPG* with the related negative controls were purchased from GenePharma (S7 Table). siRNA transfection was carried out using GenMute siRNA Transfection Reagent (SignaGen) using protocols recommended by the manufacturer.

### Establishment of stable CRC cell lines with *LIPG* knockdown or overexpression

The shRNA oligonucleotides targeting *LIPG* (S7 Table) were designed using web-based tools (https://portals.broadinstitute.org/gpp/public/gene/search). The shRNAs were inserted between the AgeI and EcoRI sites of the pLKO.1-puro vector. We produced lentivirus by transfecting pLKO.1-*LIPG*-shRNA or pLKO.1-scramble plasmids (control), packaging plasmid psPAX2 and envelope plasmid pMD2.G with LipoD293 (SignaGen) into HEK293T cells according to the manufacturer’s protocols and ultimately, used it to transduce CRC cells. Polybrene (Sigma) was used to enhance the efficiency of the lentiviral transduction, yielding a final concentration of 10μg/mL. After 24 h, the virus-containing medium was removed and replaced with fresh medium along with puromycin (4μg/mL; Sigma). After 3 days of antibiotic selection, non-infected cells were all killed but cells that were successfully transduced survived. We then checked the transduction efficiency via qRT-PCR and immunoblotting.

The full length cDNAs of *LIPG* were cloned between the EcoRI and XhoI sites of the pCDH-CMV-MCS-EF1-copGFP-T2A-Puro vector, and we used an empty vector as the control. The preparation of lentivirus and cell transduction were similarly processed as described above. The primers for amplifying the full-length cDNA of *LIPG* gene were listed in S7 Table.

### Cell proliferation assays

The processed cells and control were trypsinized, counted, and seeded in 96 well microplates (2,000 cells in 100 μL of cell suspension per well). Each sample had five replicates and were incubated for a total of 5 days. Cell proliferation was assessed with CCK-8 assays (Boster) at designated time points according to protocols recommended by the manufacturer.

### Cell migration and invasion assays

The undersides of Transwell filters (8.0 μm; Corning Costar) were coated with 20 μL human plasma fibronectin (2 μg/mL; Millipore), overnight at 4°C. For cell migration assays, the cells were trypsinized, counted, and plated onto the upper chambers (1×10^5^ per chamber) in a serum-free medium, then cultured with 600 μL complete medium for an indicated time. The chambers were put into 600 μL 4% paraformaldehyde for 15 min and stained with crystal violet (Beyotime) for 5 min. The dyed chambers were imaged with a microscope. Migrated cells were quantified by washing the chambers with 33% glacial acetic acid followed by measuring the absorbance at 450 nm with a microplate reader. For cell invasion assays, except for precoating the Transwell insert with 50 μL of Matrigel diluted by serum free medium (1:50; BD Biosciences), the other procedures were the same as the migration assays.

### Evaluation of clinical significance of *LIPG* expression

Using qPCR, we detected the *LIPG* mRNA expression of 32 matched CRC and normal tissue cDNA samples. Several independent CRC clinical datasets that are publicly available were also analyzed, such as the Oncomine Gene Expression Array Database (www.oncomine.org), TCGA and GEO Database. The correlation analysis of *LIPG* expression and clinicopathologic parameters of CRC patients were performed in the 379 CRC patients sampled from TCGA.

### Effect of pitavastatin on CRC cell viability and *LIPG* mRNA expression

CRC cells were trypsinized and split into 96-well plates (2000/well). Plated cells were treated with increasing concentrations of pitavastatin for 48 h. The cells’ viability was evaluated using CCK-8 assays (Boster) as described above. We calculated the IC_50_ using GraphPad Prism 6. Plated cells were treated with increasing concentrations of pitavastatin for increasing numbers of days (24, 48, 72 and 96 h) and underwent CCK-8 assays. CRC cells were trypsinized and split into 6-well plates. Plated cells were treated with 10 μM pitavastatin for five time periods (1, 3, 6, 9 and 12 h) and then measured their respective *LIPG* mRNA levels by qPCR.

### Statistical analysis

Functional annotation for CRC susceptibility SNPs was performed using the R software. Statistical analyses were performed using GraphPad Prism 6 and SPSS (v19.0). Linear regression analysis was performed to evaluate the association of *LIPG* expression with rs7229639 genotypes in HapMap Asian populations (CHB and JPT). Two-tailed Student’s t tests were used to assess the statistical significance of luciferase reporter assays, qPCR results for gene expression, effects of pitavastatin, as well as the results of cell proliferation, migration and invasion assays. Paired Student’s t tests were performed to examine gene expression differences between CRC and the matched adjacent normal tissues. Kaplan–Meier survival analyses were performed with the Log-rank test. We analyzed the correlation between *LIPG* expression and clinicopathologic parameters by Spearman’s correlation. Gene expression correlation analysis of the GSE97023 (n = 34) was also performed by Spearman’s correlation. *P* value less than or equal to 0.05 is considered statistically significant. * *P* ≤ 0.05, ** *P* < 0.01, *** *P* < 0.001, **** *P* < 0.0001.

## Supporting information

S1 Fig The screening and LD analysis process of CRC-related genetic variants. (PDF)

S2 Fig The A allele of rs7229639 was associated with increased *LIPG* expression. (PDF)

S3 Fig Hi-C interaction map in small bowel tissues. (PDF)

S4 Fig The tissue-specific histone modification profiles of the functional SNP region at 18q21.1. (PDF)

S5 Fig Candidate functional SNPs-mediated disruption of transcription factor motifs. (PDF)

S6 Fig *LIPG* expression at mRNA and protein levels in different CRC cell lines. (PDF)

S7 Fig *LIPG* knockdown with another siRNA sequence inhibits cell proliferation, migration and invasion in DLD-1 cells. (PDF)

S1 Table The significant eQTL SNPs in sigmoid and transverse colon tissues from GTEx. (XLSX)

S2 Table CRC-related loci associated with chromatin accessibility. (XLSX)

S3 Table CRC-associated loci overlapped with histone modification peaks. (XLSX)

S4 Table CRC-related SNPs that disrupt TF binding. (XLSX)

S5 Table The potential effects prediction of the missense SNPs using SIFT and Polyphen-2. (XLSX)

S6 Table Correlation analysis between *LIPG* expression and clinicopathological characteristics of colorectal cancer. (XLSX)

S7 Table Primers or sgRNA sequence used in this study. (XLSX)

S1 Data Numerical original data. (XLSX)
