# Supplementary material for: Identification of potential functional variants and genes at 18q21.1 associated with the carcinogenesis of colorectal cancer
Source: PLoS Genet. 2022 Feb 2;18(2):e1010050. doi: 10.1371/journal.pgen.1010050 (PMC8870576; doi:10.1371/journal.pgen.1010050)
Supplement: S1 Fig — (PDF) [file pgen.1010050.s001.pdf]

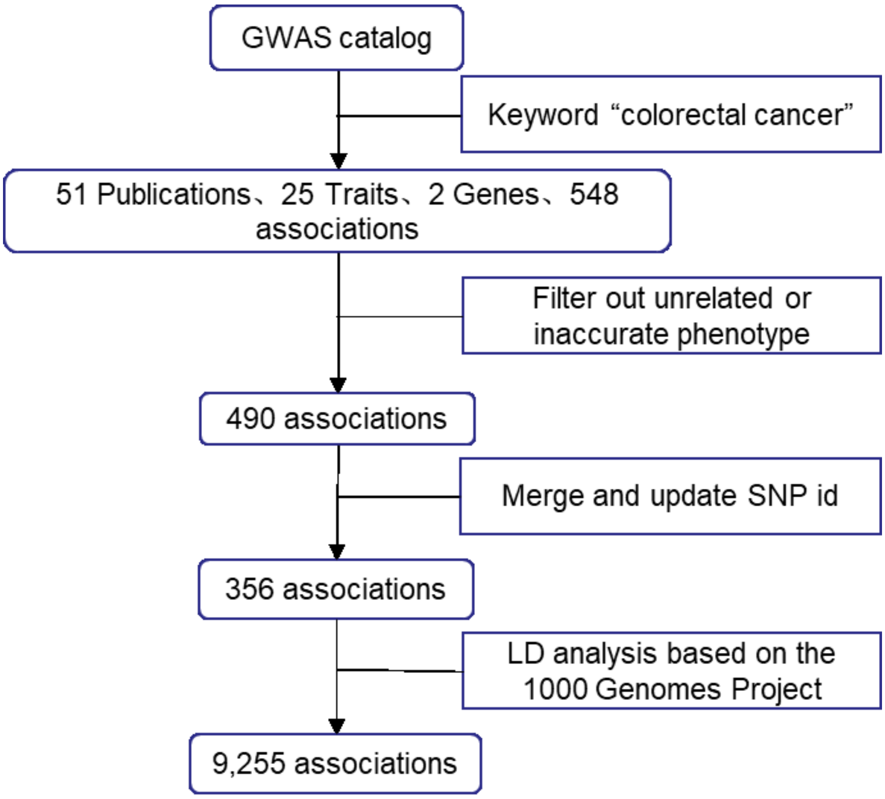

S1 Fig. The screening and LD analysis process of CRC-related genetic variants. CRC-related risk variants were obtained from GWAS catalog. Some irrelevant or imprecise phenotype (e.g., Cancer, severe skin toxicity response to cetuximab in colorectal cancer, etc.) were filtered out. SNPs that were reported repeatedly were merged, and were updated with the SNP IDs according to the latest dbSNP database. And LD calculation was performed to obtain all the SNPs in high LD ($r^2 > 0.5$) with the reported leading SNPs by using the 1000 Genomes Project data.
