# Supplementary material for: Identification of potential functional variants and genes at 18q21.1 associated with the carcinogenesis of colorectal cancer
Source: PLoS Genet. 2022 Feb 2;18(2):e1010050. doi: 10.1371/journal.pgen.1010050 (PMC8870576; doi:10.1371/journal.pgen.1010050)
Supplement: S2 Fig — (PDF) [file pgen.1010050.s002.pdf]

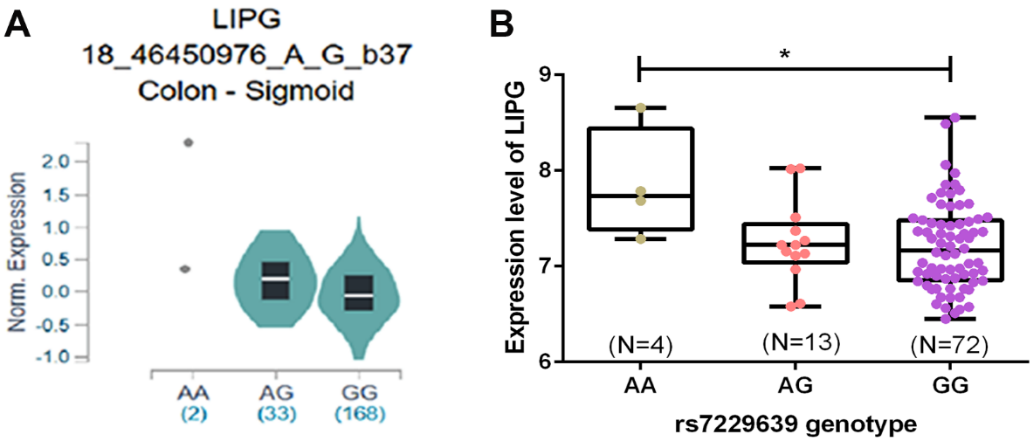

S2 Fig. The A allele of rs7229639 was associated with increased *LIPG* expression. (**A**) The eQTL data of sigmoid colon tissues from the GTEx database showed that *LIPG* expression is gradually elevated as the number of A allele (risk allele) of rs7229639 increases. (**B**) Similar trends of the increased number of A alleles and the increased expression of *LIPG* were observed in the HapMap Asian populations (CHB and JPT). Expression data were extracted from GSE6536. Linear regression analysis was used to calculate p values. * $P < 0.05$
