# Supplementary material for: Identification of potential functional variants and genes at 18q21.1 associated with the carcinogenesis of colorectal cancer
Source: PLoS Genet. 2022 Feb 2;18(2):e1010050. doi: 10.1371/journal.pgen.1010050 (PMC8870576; doi:10.1371/journal.pgen.1010050)
Supplement: S3 Fig — (PDF) [file pgen.1010050.s003.pdf]

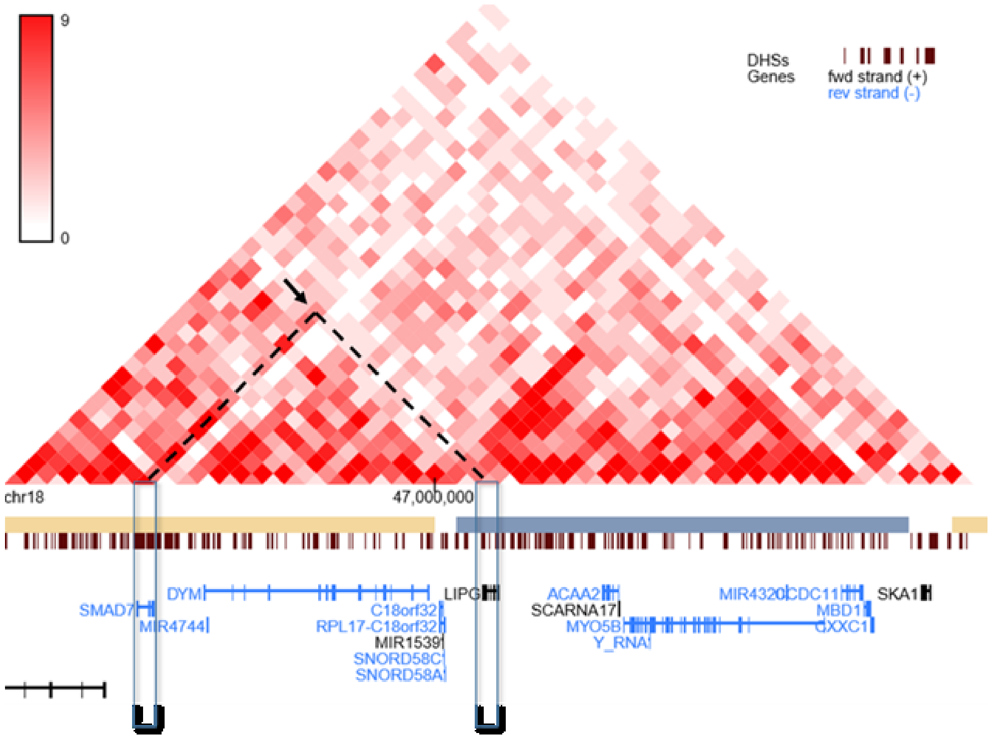


**S3 Fig. Hi-C interaction map in small bowel tissues.** The black arrow points to a potential interaction between the *SMAD7* locus and *LIPG*. The data were visualized by 3D Genome Browser (http://3dgenome.fsm.northwestern.edu/).
