# Supplementary material for: Identification of potential functional variants and genes at 18q21.1 associated with the carcinogenesis of colorectal cancer
Source: PLoS Genet. 2022 Feb 2;18(2):e1010050. doi: 10.1371/journal.pgen.1010050 (PMC8870576; doi:10.1371/journal.pgen.1010050)
Supplement: S4 Fig — (PDF) [file pgen.1010050.s004.pdf]

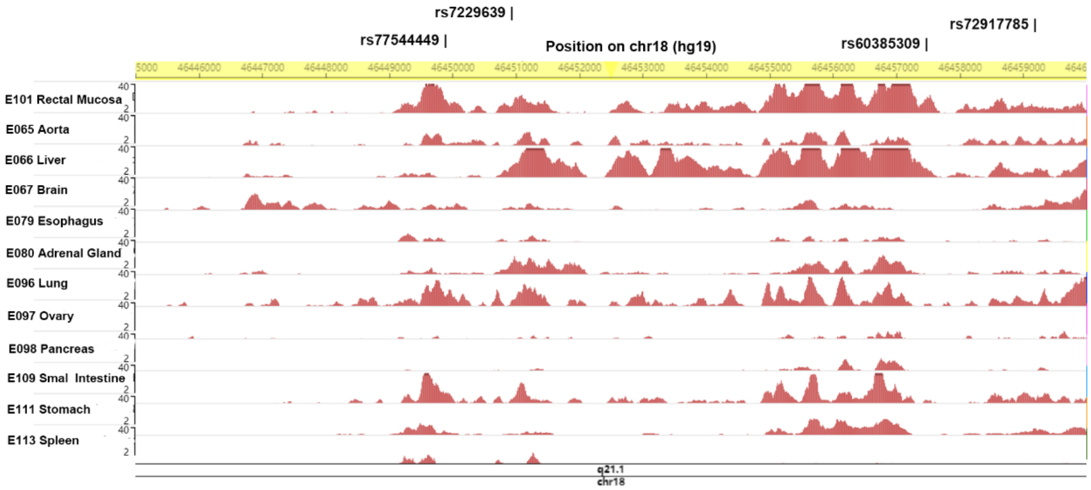

S4 Fig. The tissue-specific histone modification profiles of the functional SNP region at 18q21.1. The profiles of H3K27ac chromatin modifications of the functional SNP region at 18q21.1 were presented, with rectal mucosa tissue and 11 other tissues, indicating the high tissue specificity of the region activity. The data were taken from the Roadmap Epigenomics Project and visualized by WashU Epigenome Browser.
