# Supplementary material for: Identification of potential functional variants and genes at 18q21.1 associated with the carcinogenesis of colorectal cancer
Source: PLoS Genet. 2022 Feb 2;18(2):e1010050. doi: 10.1371/journal.pgen.1010050 (PMC8870576; doi:10.1371/journal.pgen.1010050)
Supplement: S5 Fig — (PDF) [file pgen.1010050.s005.pdf]

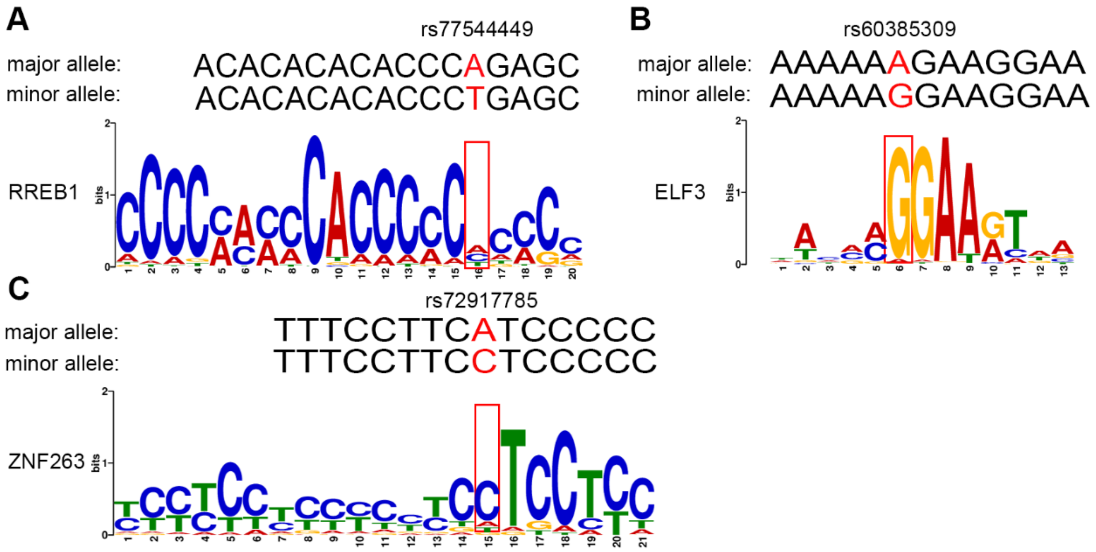

S5 Fig. Candidate functional SNPs-mediated disruption of transcription factor motifs. (**A**)The motif analysis indicated that RREB1 motif preferably bound to A allele of rs77544449. (**B**) The motif analysis indicated that ELF3 motif preferably bound to G allele of rs60385309. (**C**) The motif analysis indicated that ZNF263 motif preferably bound to C allele of rs72917785.
