# Supplementary material for: Identification of potential functional variants and genes at 18q21.1 associated with the carcinogenesis of colorectal cancer
Source: PLoS Genet. 2022 Feb 2;18(2):e1010050. doi: 10.1371/journal.pgen.1010050 (PMC8870576; doi:10.1371/journal.pgen.1010050)
Supplement: S6 Fig — (PDF) [file pgen.1010050.s006.pdf]

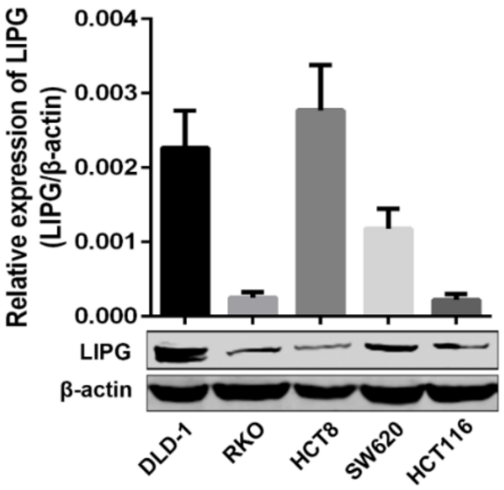

S6 Fig. *LIPG* expression at mRNA and protein levels in different CRC cell lines. *LIPG* expression was relatively high in DLD-1 and SW620 cells, and relatively low in RKO and HCT116 cells.
