# Supplementary material for: Identification of potential functional variants and genes at 18q21.1 associated with the carcinogenesis of colorectal cancer
Source: PLoS Genet. 2022 Feb 2;18(2):e1010050. doi: 10.1371/journal.pgen.1010050 (PMC8870576; doi:10.1371/journal.pgen.1010050)
Supplement: S7 Fig — (PDF) [file pgen.1010050.s007.pdf]

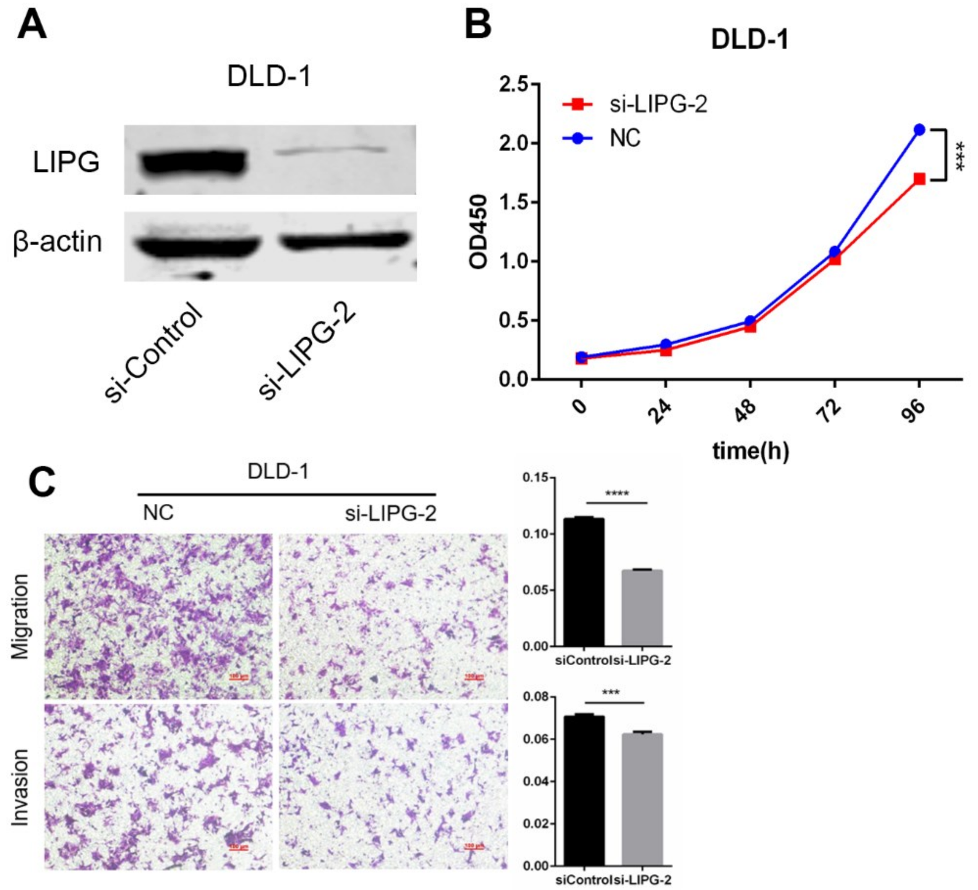

S7 Fig. *LIPG* knockdown with another siRNA sequence inhibits cell proliferation, migration and invasion in DLD-1 cells. (**A**) Validation of *LIPG* knockdown efficiency by immunoblotting analysis in DLD-1 cells. (**B**) The effect of *LIPG* knockdown on cell proliferation was measured by CCK-8 assay. (**C**) The effect of *LIPG* knockdown on cell migration and invasion capacity was measured by Transwell assay. Error bars, SD. $n = 3$. Two-tailed Student's t tests were used to assess statistical significance. ** $P < 0.01$, *** $P < 0.001$, **** $P < 0.0001$
